# Evaluation of a Web-Based Social Network Electronic Game in Enhancing Mental Health Literacy for Young People

**DOI:** 10.2196/jmir.2316

**Published:** 2013-05-15

**Authors:** Tim MH Li, Michael Chau, Paul WC Wong, Eliza SY Lai, Paul SF Yip

**Affiliations:** ^1^Department of Social Work and Social AdministrationThe University of Hong KongHong KongChina (Hong Kong); ^2^HKJC Centre for Suicide Research and PreventionThe University of Hong KongHong KongChina (Hong Kong); ^3^School of BusinessThe University of Hong KongHong KongChina (Hong Kong)

**Keywords:** digital game-based learning, mental health literacy, social networking sites, motivation

## Abstract

**Background:**

Internet-based learning programs provide people with massive health care information and self-help guidelines on improving their health. The advent of Web 2.0 and social networks renders significant flexibility to embedding highly interactive components, such as games, to foster learning processes. The effectiveness of game-based learning on social networks has not yet been fully evaluated.

**Objectives:**

The aim of this study was to assess the effectiveness of a fully automated, Web-based, social network electronic game on enhancing mental health knowledge and problem-solving skills of young people. We investigated potential motivational constructs directly affecting the learning outcome. Gender differences in learning outcome and motivation were also examined.

**Methods:**

A pre/posttest design was used to evaluate the fully automated Web-based intervention. Participants, recruited from a closed online user group, self-assessed their mental health literacy and motivational constructs before and after completing the game within a 3-week period. The electronic game was designed according to cognitive-behavioral approaches. Completers and intent-to-treat analyses, using multiple imputation for missing data, were performed. Regression analysis with backward selection was employed when examining the relationship between knowledge enhancement and motivational constructs.

**Results:**

The sample included 73 undergraduates (42 females) for completers analysis. The gaming approach was effective in enhancing young people’s mental health literacy (*d*=0.65). The finding was also consistent with the intent-to-treat analysis, which included 127 undergraduates (75 females). No gender differences were found in learning outcome (*P*=.97). Intrinsic goal orientation was the primary factor in learning motivation, whereas test anxiety was successfully alleviated in the game setting. No gender differences were found on any learning motivation subscales (*P*>.10). We also found that participants’ self-efficacy for learning and performance, as well as test anxiety, significantly affected their learning outcomes, whereas other motivational subscales were statistically nonsignificant.

**Conclusions:**

Electronic games implemented through social networking sites appear to effectively enhance users’ mental health literacy.

## Introduction

The Internet plays an increasingly important role in our daily lives. With the shift of the World Wide Web from Web 1.0 to Web 2.0, the Web becomes an essential platform for information sharing, user-centered design, and collaboration. The Web 2.0 concept has led to the development and evolution of Web-based communities and applications, such as social networking sites (SNS), video-sharing sites, blogs, forums, wikis, and interactive games.

Apart from leisure use, some studies have indicated that the Internet has become an instrumental information-searching tool for people with health concerns [1,2], especially for adolescents [3,4]. Informative educational websites containing physical and mental health information, such as the National Institutes of Health, Beacon Health, and beyondblue [5-7] are examples of contemporary health education initiatives. The Internet has been proposed to be an efficient platform to engage, educate, and intervene in the younger generation’s health concerns, especially for those who are not easily engaged by traditional methods [8,9]. Previous literature suggested that Internet-based intervention on health education for adolescents was more effective than for adults [9]. There are some interventions primarily focused on providing mental health education, such as Kindertelefoon, YooMagazine, and ReachOut [10-12]. Those interventions engage young people through interactive forums, games, and websites to enhance their mental health literacy.

Different kinds of multimedia techniques and interaction methods based on advanced technologies have been evaluated for their learning efficacy through preliminary evidence [13-15]. In particular, the potential benefits of using digital games for instructional purposes are worthy of investigation. Many studies have shown the benefits and educational effectiveness of digital game-based learning (DGBL) in both classroom and laboratory settings, and in various subjects like computer science, engineering, mathematics, and physics [16-20]. The literature identifies motivational characteristics of educational computer games and also establishes characteristics that consistently facilitate game design and development [21-24]. Those characteristics encompass the use of a fantasy environment, scenarios that engage curiosity, present challenges, and are dependent on the player’s control. It is believed that the learning experience can be enriched by those characteristics. For example, adding gaming features like fantasy and mastery could make learning more interesting and enjoyable [25,26], and electronic games could enhance children’s and adolescents’ knowledge, attitudes, and health behaviors as effectively, or even more, than means of conventional instruction [19]. In addition, the game environment is not only goal-directed and rule-governed, but also contains interactive elements (eg, competition), whether it is intended for one or multiple players [21,27]. Furthermore, literature suggests that games can foster high interactivity and experiential learning, improving players’ health-related self-efficacy and behaviors; engage young people who are difficult to influence through traditional health education interventions; provide supportive and informative feedback on health choices; support self-paced progress; offer opportunities for social interaction and health-related social support (both within the game and around it), increasing players’ motivation towards improving health behaviors; and offer opportunities to rehearse self-care skills, which can be applied in real-life situations [19,28]. Nonetheless, the effectiveness of DGBL in mental health education has been underexplored. In merging the findings and benefits of DGBL in health education, it is worthwhile to examine the effects of DGBL on mental health education.

In Hong Kong, mental health education is not a regular school subject in the school curriculum. Rather, some concepts and skills may be taught in subjects like Life Education or Liberal Studies. Therefore, some school-based mental health promotion programs were developed. For instance, “The Little Prince is Depressed” project was a 12-week, school-based, universal program aimed at reducing depressive symptoms and enhancing protective factors of depression among secondary school students [29]. Its curriculum was developed based on the cognitive-behavioral model and included topics like stress and depression, and cognitive restructuring and problem-solving skills. Students showed positive development in help-seeking attitudes and self-esteem in general, and those with more depressive symptoms significantly improved their cognitive restructuring skills and support-seeking behaviors after participating. However, the number of beneficiaries of school-based programs is limited to the resources available, and competition for school teaching hours to carry out such a program is significant. Therefore, a new form of intervention to promote mental health among young people is necessary in Hong Kong.

In the past few years, online social networks, an important aspect of Web 2.0 applications, have emerged as a mainstream communication and interaction modality, especially among young people. SNS, such as Facebook, can be an excellent platform to reach a large spectrum of the population. People make use of these platforms to have fun and socialize with each other through games, chats, and status updates. Games on social networks are called “social games” and attract large numbers of players every day. For example, many people, especially adolescents, spend a lot of time growing crops on “Happy Farm” and feeding pets in “Pet Society” on Facebook. This new form of communication modality, however, has created both concerns and opportunities for health professionals and researchers. For instance, some reported that SNS might be related to problematic use of the Internet [30], whereas others found SNS to be a more useful tool for procuring social support [31] and improving participants’ engagement and retention in Web-based interventions [32]. Nevertheless, Facebook has been regarded as a good platform to reach and educate young people, with its large number of members across the world. Studies concerning interventions with the help of Facebook have emerged recently [32,33].

Previous studies revealed gender differences in the use of Internet. For example, males have been shown to spend more time playing games and on other forms of entertainment, whereas females use the Internet more for communication and SNS [34,35]. Therefore, it is important to investigate whether gender plays a role in DGBL on Internet SNS.

The current study attempted to evaluate a novel approach of using DGBL through an SNS for mental health education. We designed a learner-centered, self-paced, electronic game, meaning that people could play and learn whenever they wanted to and could achieve as much as they preferred in terms of domain knowledge enhancement. The game developed for this study was placed on Facebook, and no facilitator was required. We hypothesized that mental health literacy would be enhanced through Web-based DGBL and three motivational constructs (ie, expectancy, value, and affect), would influence learning outcomes. Mental health literacy was first defined as “knowledge and beliefs about mental disorders which aid their recognition, management or prevention” (page 182, [36]). Here, the definition of mental health literacy not only included knowledge of a mental disorder (ie, depression), but also knowledge related to mental health problems, such as stress, its management, and important skills that might be helpful for one’s mental health (eg, cognitive restructuring skills and problem-solving skills). Beliefs about mental disorders were beyond the scope of current study. Specifically, we sought to:

1. explore the effectiveness of using DGBL to enhance mental health literacy through an SNS;
2. explore learning motivation in DGBL;
3. examine whether gender plays a role in intervention effectiveness and learning motivation; and
4. explore the impact of motivational constructs on mental health literacy.

## Methods

### Participants

Participants were recruited at a major university in Asia. An invitation email was sent to all undergraduate and postgraduate students’ (N=22,260) university email accounts inviting them to participate in the current study. Students who were between 17-25 years old, had adequate Internet literacy and a Facebook account, and were reachable via the local network were eligible to participate. The invitation email explained to participants that they would not only be required to complete a 3-week game on Facebook, but would also be asked to complete a set of Web-based, self-assessed questionnaires both before and after finishing the game. They were allowed to play the game anytime and anywhere with a computer connected to the Internet within the study period. Those who completed the game and the online questionnaires received cash compensation and were entered in a drawing for a tablet computer, supermarket coupons, and theme park tickets. Interested students replied to the invitation email and provided their Facebook ID for verification. The research team then confirmed their eligibility through email.

### The Game: “Ching Ching Story”

#### Theoretical Background

The electronic game “Ching Ching Story” and its content were developed by the authors of the paper. The development process involved contextual and technical aspects. For the contextual aspect, the learning content was adopted and modified from a school-based, mental health enhancement program for adolescents by members of the research team. The school-based program effectively reduced depressive symptoms within those with highly depressed moods [29]. Its content was developed based on a cognitive-behavioral therapeutic approach, which is consistent with diathesis-stress models of depression. Such models emphasize the cognitive and behavioral characteristics of an individual, which not only affect the impact of adverse life events, but also have a direct influence upon the development of depression. Cognitive-behavioral therapy is an evidence-based treatment approach for various mental health problems, such as depression and anxiety [37,38].

“Ching Ching Story” consisted of 10 topics: (1) identifying stressors and how to handle stress, (2) understanding the relationship between stress and coping, and the consequence of depression, (3) understanding what goal-directed thinking is, (4) affirming existing strengths and acknowledging the concept of “self”, (5) cognitive restructuring, (6) advanced cognitive restructuring, (7) understanding others’ feelings, (8) communication skills, (9) conflict resolution based on a problem-solving approach, and (10) anger management [29].

#### Technology

**Figure 1:**
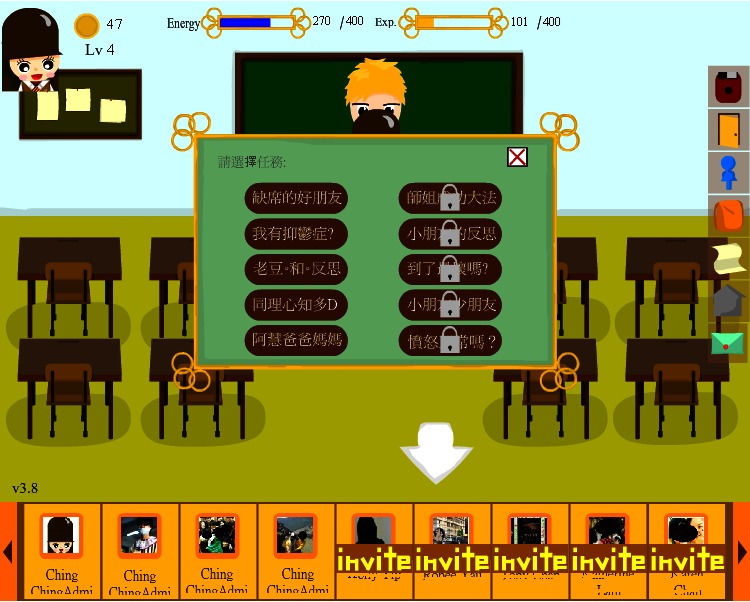
Missions for players to choose.

**Figure 2:**
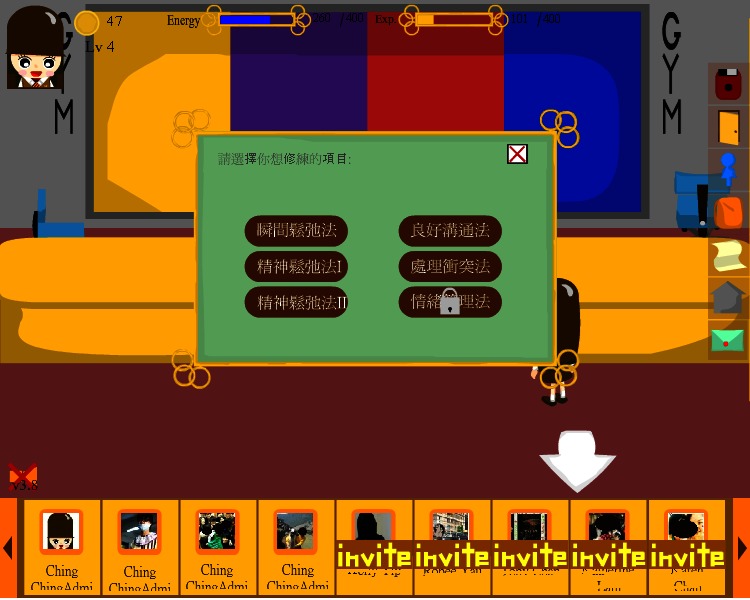
Psychological gym room.

**Figure 3:**
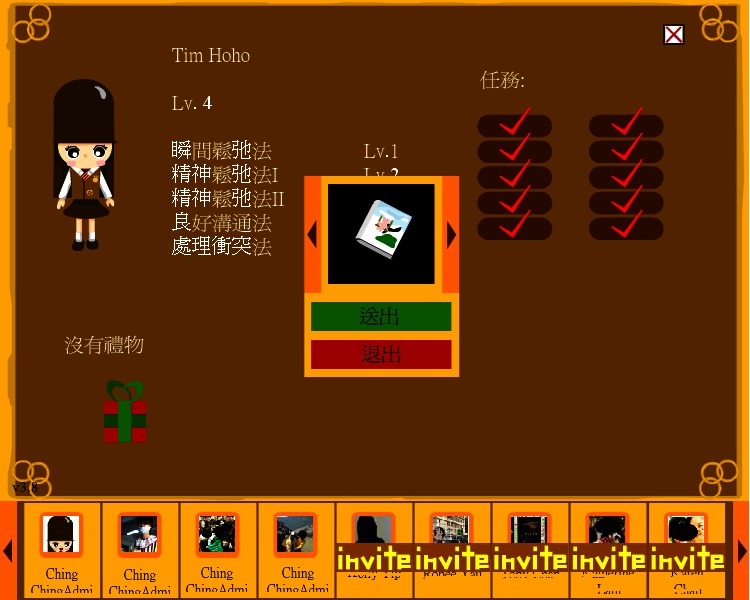
Visiting friends and sending gifts.

Adobe Flash was used to produce interactive gaming elements and animated graphics on the client side of the system. Flash ActionScript 3.0 handled the logic of the game and sent requests to the backend in response to user actions. Moreover, we used Facebook API, where both Facebook iFrame and Javascript SDK facilitated the retrieval of a user’s profile and social network data from the Facebook application. Javascript was used for backend programming. Questionnaire and game progress data were stored in a MySQL database. The system was hosted on a computer server on the authors’ host institution network. The game was accessible by the public on Facebook [39]. Three screenshots of the game are shown in Figures 1-3.

#### Structure

“Ching Ching Story” is a role-playing game. It is thought that proper instructional design is capable of facilitating both mental health learning and intervention [40]. The game adopted a problem-based, narrative, adventure approach. Players assumed the role of the game character, Ching Ching, and moved around different areas of the game to complete all missions by talking to nonplayer characters, exploring different places and objects, and playing various minigames, which taught instrumental skills. The whole game consisted of ten missions to be fulfilled as shown in Figure 1 with a storyline. Each mission incorporated different mental health concepts. Some of the missions were expected to be either more challenging or required prior knowledge, which was learned in other missions. As shown in Figure 2, after participants successfully completed particular tasks in the “psychological gym room”, some skills, such as ones within the communication domain, could become part of Ching Ching’s abilities. Such abilities were required to complete other tasks, thereby encouraging participants to recall those important skills more often throughout the game. Ching Ching’s “energy” was consumed after working on some tasks, and when Ching Ching consumed all possible energy, the player could not progress and advance the plot until energy was recovered. This aspect was incorporated in order to avoid players completing the whole game in a very short time, which might have deterred effective learning.

Despite the limitations on playtime, players still had great flexibility within the game, selecting the mission they preferred. In an effort to encourage active and self-paced learning, there was no predefined order of completion. Once players completed the ten missions, no advanced plots and/or tools were provided. Also, a level system was installed to record the level and skills learned for each player. The record was shared among friends who also joined the game to establish a leader board and create an atmosphere of competition to enhance the game’s appeal. Moreover, the game facilitated social support by encouraging interaction between players. Examples included sending gifts and greetings among friends, shown in Figure 3, which served to increase player retention. Since some tasks required special tools to accomplish, players could help each other by sending the tools as gifts. Moreover, to encourage players to invite their friends to join the game, gifts were offered to players who sent more invitations to play the game. “Level-up” and “task completed” notifications were posted on players’ Facebook walls to acknowledge their achievements. The players’ friends therefore knew of, and could comment on, players’ progress and achievements. The research team provided technical support in case participants encountered any technical problems while playing the game. Apart from this, no other intervention or support was provided. Finally, no prompts or reminders were used in the game to maintain gameplay frequency or duration.

### Outcome Measures

#### Overview

Two sets of Web-based, self-assessed questionnaires were developed for measuring participants’ mental health literacy (primary outcome) and their learning motivation (secondary outcome). Facebook ID was used as a user identifier for verification and avoided duplicate entries. Pre- and posttests automatically popped up once the game started and ended, respectively. The pretest consisted of demographic information and self-developed questions on mental health literacy, whereas the posttest consisted of the same questions for mental health literacy plus modified questions for learning motivation from the Motivated Strategies for Learning Questionnaire (MSLQ) [41].

#### Mental Health Literacy

Mental health literacy was measured through 31 questions, which were constructed by the researchers to address the 10 topics of the game. They included true-or-false, sequencing, multiple-choice, and matching questions. The questions, designed to evaluate a broader understanding of learning through online DGBL, covered topics like understanding of mental health concepts and application of skills. Sample questions may be found in Multimedia Appendix 1.

#### Learning Motivation

Participants’ learning motivation was measured in terms of (1) *value*: intrinsic goal orientation (4 items), extrinsic goal orientation (4 items), task value (6 items), (2) *expectancy*: control of learning beliefs (4 items), self-efficacy for learning and performance (8 items), and (3) *affect*: test anxiety (5 items). Reliability and predictive validity of the MSLQ were properly evaluated in previous studies [41]. All items were rated on a 7-point Likert scale (1 = *not at all true for me*; 7 = *very true of me*). The list of items used is shown in Multimedia Appendix 1.

### Statistical Methods

Descriptive statistics were used to illustrate the general picture of the data for each measurement. Any participants with missing data or who dropped out were not included in the completers analysis. In addition to completers analysis, data were analyzed through an intent-to-treat (ITT) analysis, using multiple imputation (MI) to address loss of follow-up data. Dropout participants who completed questionnaires only at the pretest were handled by applying the technique of MI with 5 imputations for missing data [42]. We conducted missing values analysis to investigate whether data were missing completely at random (MCAR). A within-subject Student’s *t*-test was used to analyze the statistical significance of changes in knowledge, and a Student’s *t*-test for independent samples was employed to test for gender differences.

Linear regression was used to examine the relation between knowledge enhancement and motivational constructs. By fitting knowledge enhancement with motivational constructs, the coefficient and 95% confidence interval could be presented. The regression was further adjusted by adding pretest score, gender, and age as control variables. Backward selection was employed in the regression analysis. All data analyses were conducted using SPSS software.

### Ethics and Informed Consent

Ethics approval, from the Human Research Ethics Committee for Non-Clinical Faculties at the authors’ institution, was obtained before data collection. Participants provided informed online consent before they started the game. The informed consent form detailed information about the procedures of the study.

## Results

### Recruitment and Subjects

**Figure 4:**
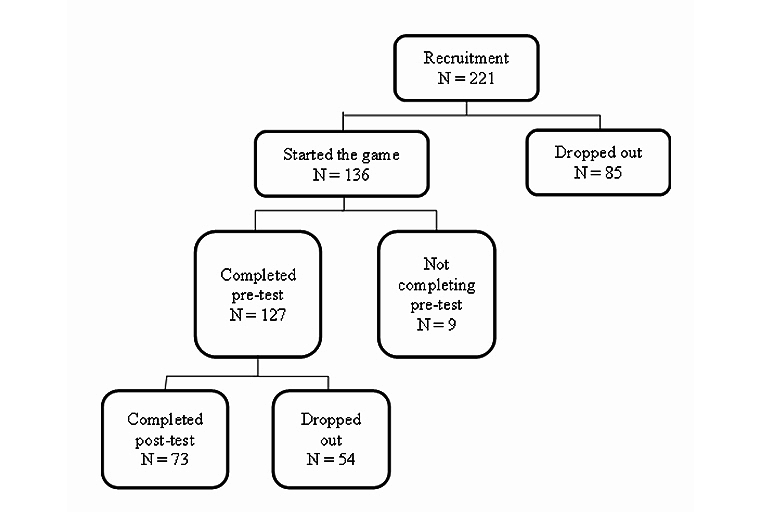
Participant flowchart.

Participants were recruited from November to December 2011 at the authors’ institution. A total of 221 undergraduates agreed to participate in the study. Out of those who started the game (n=136), a majority completed the pretest (n=127). The other 9 participants did not fully complete the pretest due to technical problems, such as early termination of the questionnaire. Out of these 127 participants, 73 completed both the pre- and posttest. All 127 participants were included in the ITT analysis, whereas only data from the 73 participants who completed both assessments were used for the completers and regression analyses. Figure 4 shows the participant flow. A total of 31 males and 42 females, ranging from 17-25 years old (M=20.82, SD 1.81), completed both assessments.

### Mental Health Literacy

Table 1 shows the number of correct responses participants achieved on the mental health literacy assessment both before and after playing the game, as well as their overall improvement levels. Both male and female participants achieved over half correct at pretest, suggesting participants’ possessed good background knowledge on mental health. The pretest scores of males and females were similar, indicating that their familiarity with mental health was comparable. The improvement of both genders was also statistically significant and consistent, revealing the effectiveness of the game play intervention for both males and females. In a *t*-test comparison between the results of the pre- and posttest, a statistically significant improvement was documented. On average, participants answered 2.21 more questions correctly after the intervention (total of 31 questions on the knowledge test). Participants demonstrated moderate improvements between pre- and posttests in terms of mental health literacy (*d*=0.65). Furthermore, there was not a statistically significant gender difference in knowledge improvement (*P*=.97). Apart from completers analysis, noncompleters were also included in the ITT analysis to provide more understanding about the effects of the intervention. Missing values analysis demonstrated that the hypothesis that the data were MCAR could not be rejected, *χ* ^2^_Little_=3.75, (*P*=.15). ITT analysis showed that the improvement between pre- and posttest was statistically significant, with a moderate effect size (*d*=0.66). This result was consistent with the completers analysis (*d*=0.65).

### Learning Motivation

Table 2 presents the descriptive statistics of the motivational subscales. *Value* focuses on the reasons why participants engaged in the educational game. Among the three subscales that made up the construct (intrinsic and extrinsic goal orientation and task value), both genders achieved the highest score on intrinsic goal orientation. On the other hand, extrinsic goal orientation was the lowest among the three value subscales. *Expectancy* refers to participants’ beliefs that they could accomplish tasks in the game. Satisfactory scores on both subscales of the expectancy construct demonstrated participants’ confidence in the acquisition of mental health knowledge. *Affect* concerns test anxiety associated with the knowledge test. It is reasonable that the anxiety scores were low in a gaming context since learning in a game should be enjoyable and relaxing. Moreover, since the test in this study was conducted online and without a time limit, test anxiety was likely alleviated. Indeed, participants’ scores on the anxiety measure were the lowest among the 6 motivational subscales. *T*-tests revealed no statistically significant gender differences on any of the 6 motivational subscales (Table 2).

### Influence of Motivational Constructs on Learning Outcomes

Nonsignificant motivational subscales were eliminated in the regression analysis with backward selection. Nonsignificance of the three subscales within the value construct indicated that reasons for participating in the game did not affect knowledge enhancement. The influence of control of learning beliefs in the expectancy construct was also not statistically significant. Control variables, namely pretest score, gender, and age, were retained in the model regardless of their level of statistical significance. As expected, gender and age did not affect learning outcomes, whereas pretest score did significantly affect knowledge enhancement (see Table 3). This result is reasonable given that pretest score restricted the maximum possible improvement on the posttest. In other words, participants attaining high scores on the pretest, when compared to those with lower scores on the pretest, could not improve as much on the posttest. Consequently, pretest score negatively influenced knowledge improvement. Furthermore, self-efficacy for learning and performance significantly affected knowledge improvement. Test anxiety, on the other hand, negatively affected knowledge enhancement.

**Table 1:** Descriptive statistics of pretest, posttest, and evidenced improvement on the knowledge test.

	Male	Female	All
Pretest score, mean (SD)	19.61 (3.30)	18.55 (3.63)	19.00 (3.51)
Posttest score, mean (SD)	21.85 (3.53)	20.74 (3.12)	21.21 (3.33)
Improvement, mean (SD)	2.23 (4.39)	2.19 (3.07)	2.21 (3.66)
*P* value of *t-*test between pre- and posttest scores	.008	<.001	<.001

**Table 2:** Descriptive statistics of the six motivational subscales and gender comparisons.

Motivational subscales		Male, mean (SD)	Female, mean (SD)	All, mean (SD)	*P* value of *t* test between genders
**Value**					
	Intrinsic goal orientation	5.02 (0.78)	4.93 (1.01)	4.97 (0.91)	.68
	Extrinsic goal orientation	4.18 (1.22)	3.71 (1.34)	3.91 (1.30)	.13
	Task value	4.74 (0.73)	4.66 (1.17)	4.70 (1.00)	.72
**Expectancy**					
	Control of learning beliefs	4.82 (0.83)	4.70 (0.93)	4.75 (0.88)	.56
	Self-efficacy for learning and performance	4.92 (0.70)	4.71 (0.92)	4.80 (0.83)	.29
**Affect**					
	Test anxiety	3.59 (1.19)	3.16 (1.23)	3.34 (1.23)	.14

**Table 3:** Regression of knowledge enhancement on motivational constructs adjusted by pretest score, gender, and age after backward selection.

Variables	Coefficients	95% CI Lower	95% CI Upper	*P* value
Self-efficacy for learning and performance	.21	0.09	0.32	<.001
Test anxiety	-.19	-0.31	-0.06	.005
Gender	.81	-0.55	2.18	.24
Age	.18	-0.20	0.56	.34
Pretest score	-.63	-0.83	-0.44	<.001

## Discussion

The fully automated Web-based intervention was effective in enhancing young people’s mental health literacy. Intrinsic goal orientation was the primary factor in learning motivation, whereas test anxiety was successfully alleviated in the game setting. No gender differences were found on any outcome measures. Moreover, self-efficacy for learning and performance positively influenced learning outcomes, whereas test anxiety negatively affected them.

### Mental Health Literacy

To our knowledge, this is one of the first studies to explore the use of DGBL through an SNS to enhance mental health literacy. Despite the limitations of this exploratory study, participants attained mental health knowledge in a continual and self-paced manner. With moderate improvements among participants, it seems players may be able to acquire knowledge in the game at their own learning pace and apply the skills to solve real-life problems. This finding provides supporting evidence that Web-based DGBL can effectively enhance mental health knowledge. The combination of gaming concepts and online education may also facilitate self-paced learning processes in higher-order thinking. It seems that this new type of learning initiative for mental health information is efficient for both male and female learners.

Thus, the current study reinforces Lieberman’s suggestion [28] that games in health education are beneficial in various aspects, including the support of a self-paced learning process. Furthermore, although the learning content of the electronic game was modified from a school-based mental health enhancement program for adolescents [29], it demonstrated its effectiveness in enhancing the mental health literacy of young people aged 17-25. Therefore, we may extend the findings to a slightly older age group on Internet-based intervention on health education. Previous literature suggested that Internet-based intervention on health education was more effective on adolescents than on adults, as such intervention for adolescents demonstrated small-to-moderate effect size, whereas similar interventions for adults usually yielded small effect size only [9]. With moderate effect size found in the current study, it is suggested that social and gaming features may enhance the effectiveness of Internet-based intervention on health education for young adults.

### Learning Motivation and Its Influence on Learning Outcomes

Participants’ learning motivation was generally positive. They believed in their own abilities to learn in the game and tailored their learning processes in the absence of a facilitator. Although participants who were outstanding in the knowledge test were rewarded with an extra prize, intrinsic goal orientation was still stronger when compared to extrinsic orientation. High intrinsic motivation indicates that participants primarily perceived that they took part in the game for its challenges, out of curiosity, and in an effort to attain mastery. Therefore, participants enjoyed playing the game and learning about mental health knowledge throughout the intervention, instead of simply pursuing rewards. With high expectancy, participants also believed that their efforts to learn throughout the self-paced game would result in positive learning outcomes. Self-directed learning was possible in the game. The positive influence of self-efficacy for learning and performance on learning outcomes implies participants’ expectations about their own performance and judgment generally reflected their learning outcomes. Participants were capable of enhancing their mental health knowledge and manage the learning process in the game. Moreover, the game-based environment provided an enjoyable learning environment for participants, evidenced by substantially reductions in their stress over the knowledge test. However, participants’ worries and concerns about the test could have still resulted in performance decrements. Although the knowledge test was conducted in a casual way, relationships between test anxiety and test results still existed.

In addition, the feedback collected from participants on learning motivation and game design provided additional information about the high intrinsic goal orientation. The feedback from participants was generally positive and encouraging. Participants appreciated the combination of gaming concepts and mental health knowledge. The transformation of mental health knowledge into a game made mental health knowledge easy to understand and attractive (eg, “The game helped me understand more about stress management”; “Overall the game was quite interesting as it used animations to show what stress and emotional distress are and solutions to manage them”). They also found that the game was good to raise their awareness in mental health (eg, “Informative about mental disorder”; “A good way to promote awareness in mental health”), and the game itself was interesting and interactive (eg, “Interactive and easy to play”). This may explain why intrinsic goal orientation remained high in the learning process. Some participants, however, pointed out the game sometimes had too many words, which may decrease the enjoyment of the game (eg, “More informative than interesting”; “Sometimes want to skip information and conversations to advance plots”). We therefore may need to think of balancing information and entertainment to achieve better learning motivation in our future intervention development.

### Limitations

There are some limitations of this exploratory study: lack of a control group, small sample size, high dropout rate, and a biased sample. Moreover, the social functions in the game might not have been fully utilized, as the total number of players was not large. Time limitations prevented the number of players in the game to grow to an adequate size. Since the number of players influences the interactivity of a social game, the game in this study might not have been as interactive as other social games for pure entertainment on the market. In addition, our DGBL study was domain-specific, a common issue in most previous studies.

### Further Research

In sum, DGBL in combination with an online social communication platform in health education should be advocated as a way to promote mental health awareness and equip people with domain-specific knowledge education, and warrants further examination. Future studies should involve a larger sample, incorporate a control group, and recruit different age groups in order to have a more comprehensive understanding of the effects of this Web-based electronic game on mental health education. In addition to augmenting the sample size, another future research direction includes investigating the effectiveness of social game education based on this study. Social games on SNS facilitate social interaction and communication, making them different from traditional online games. SNS could provide tools to embed communication platforms (eg, blogs, forums) into games, which may, in turn, substantially enhance levels of sharing and knowledge exchange among players [43]. These social elements can motivate players to learn and potentially foster the learning process. DGBL should not be restricted to learning inside the game but should extend outside the realm of the game (eg, discussing learning material on blogs or forums). The effectiveness of social game education needs further evaluation. Also, the scope of this research should be extended to different health issues, such as mood and eating disorders. Through such future work, DGBL can be fully utilized and evaluated in different aspects of health education.

## Multimedia Appendix 1

Questionnaire items.

## Multimedia Appendix 2

CONSORT-EHEALTH checklist V1.6.2 [44].

## Abbreviations

DGBL: digital game-based learning
ITT: intent-to-treat
MCAR: missing completely at random
MI: multiple imputation
MSLQ: Motivated Strategies for Learning Questionnaire
SNS: social networking sites
