# Supplementary material for: Evaluation of a Web-Based Social Network Electronic Game in Enhancing Mental Health Literacy for Young People
Source: J Med Internet Res. 2013 May 15;15(5):e80. doi: 10.2196/jmir.2316 (PMC3668612; doi:10.2196/jmir.2316)
Supplement: Supplementary file 1 [file jmir_v15i5e80_app1.pdf]

Multimedia Appendix 1. Questionnaire Items.

Sample questions for mental health literacy:

Section A: Knowledge of mental disorders and skills for promoting mental health (True or False)

1. Depression is a mood disorder.
2. People with sadness will feel neverending unhappiness.
3. Depression weakens our self-confidence.

Section B: Rearranging the 6 goal-setting steps in correct order

a. Evaluate the progress
b. Set a goal that can reflect your value
c. Evaluate pros and cons of each method
d. Brainstorming
e. Understand your value
f. Implement your plan

Section C: Identifying thinking errors in given scenarios. Options included: (1) overgeneralization; (2) black-or-white thinking; (3) jumping to conclusion; (4) magnification/minimization; (5) catastrophization; and (6) personalization.

Scenario 1: Kitty said, "I know Mr. Chan doesn't like me, I just know."
Scenario 2: Gillian's parents are going to get a divorce, so she decided not to get married because she thought her marriage would not last long.

Section D: Identifying common ways of conflict resolution: dominance; compromise; avoidance; accommodation; and collaboration.

Modified questions for learning motivation from Motivated Strategies for Learning Questionnaire (MSLQ):

Motivational subscales	Question number
**Value construct**	
Intrinsic Goal Orientation	1, 16, 22, 24
Extrinsic Goal Orientation	7, 11, 13, 30
Task Value	4, 10, 17, 23, 26, 27
**Expectancy construct**	
Control of Learning Beliefs	2, 9, 18, 25
Self-Efficacy for Learning and Performance	5, 6, 12, 15, 20, 21, 29, 31
**Affect construct**	
Test Anxiety	3, 8, 14, 19, 28

The rating scale was as follows:

1 2 3 4 5 6 7

(1 = *not at all true for me*; 7 = *very true of me*)

1. In a game like this, I prefer mental health material that really challenges me so I can learn new things.
2. If I study in appropriate ways, then I will be able to learn the mental health material in this game.
3. When I complete a task in the game/item on the questionnaire, I think about how poorly I am doing compared to other players.
4. I think I will be able to use what I learn in this game in my life.
5. I believe I will receive an excellent result on the following quiz.
6. I'm certain I can understand the most difficult mental health material presented in the tasks for this game.
7. Getting a result on the following quiz/tasks in the game is the most satisfying thing for me right now.
8. When I complete a task/quiz, I think about items on other parts of the task/quiz I can't answer.
9. It is my own fault if I don't learn the mental health material in this game.
10. It is important for me to learn the mental health material in this game.
11. The most important thing for me right now is improving my overall result, so my main concern in this game is getting a good result on the following quiz/tasks in the game.
12. I'm confident I can learn the basic concepts taught in this game.
13. If I can, I want to get better results on the following quiz/tasks in the game than most of the other players.
14. When I complete tasks/quizzes I think of the consequences of failing.
15. I'm confident I can understand the most complex mental health material presented by this game's tasks.
16. In a game like this, I prefer mental health material that arouses my curiosity, even if it is difficult to learn.
17. I am very interested in the content area of this game.
18. If I try hard enough, then I will understand the mental health material.
19. I have an uneasy, upset feeling when I complete tasks/quizzes.
20. I'm confident I can do an excellent job on the tasks in this game.
21. I expect to do well in this game.
22. The most satisfying thing for me in this game is trying to understand the content as thoroughly as possible.
23. I think the mental health material in this game is useful for me to learn.
24. When I have the opportunity in this game, I choose tasks that I can learn from, even if they don't guarantee a good result on subsequent quizzes/tasks in the game.

25. If I don’t understand the mental health material, it is because I didn't try hard enough.
26. I like the subject matter of this game.
27. Understanding the subject matter of this game is very important to me.
28. I feel my heart beating fast when I complete tasks/quizzes.
29. I’m certain I can master the skills being taught in this game.
30. I want to do well in this game because it is important to show my abilities to my family, friends, employer, or others.
31. Considering the difficulty of this game, the tasks, and my skills, I think I will do well in this game.
