# Supplementary material for: Evaluation of a Web-Based Social Network Electronic Game in Enhancing Mental Health Literacy for Young People
Source: J Med Internet Res. 2013 May 15;15(5):e80. doi: 10.2196/jmir.2316 (PMC3668612; doi:10.2196/jmir.2316)
Supplement: Supplementary file 2 [file jmir_v15i5e80_app2.pdf]

CONSORT-EHEALTH Checklist V1.6.2 Report

(based on CONSORT-EHEALTH V1.6), available at [http://tinyurl.com/consort-ehealth-v1-6].

2316

Date completed

1/24/2013 21:31:30

by

Tim Li

Evaluation of a Web-based Social Network Electronic Game in Enhancing Mental Health Literacy for Young People

TITLE

1a-i) Identify the mode of delivery in the title

The words "web-based" and "electronic game" in the title of the study were used to highlight the nature of the research context. Since the game in the study was an offline game, "social network electronic game" was used to clearly describe the nature of the game.

1a-ii) Non-web-based components or important co-interventions in title

1a-iii) Primary condition or target group in the title

The words "young people" in the title of the study were used to highlight the target group of the study.

ABSTRACT

1b-i) Key features/functionalities/components of the intervention and comparator in the METHODS section of the ABSTRACT

The research design was stated that "The pre-test–post-test design is used in the study to evaluate the intervention". The components of the intervention was mentioned for instance "The electronic game was designed based on a cognitive-behavioral approach".

1b-ii) Level of human involvement in the METHODS section of the ABSTRACT

The word "fully automated" was used to describe the level of human involvement in the METHODS section of the ABSTRACT.

1b-iii) Open vs. closed, web-based (self-assessment) vs. face-to-face assessments in the METHODS section of the ABSTRACT

We clearly stated that "Participants recruited from a closed online user group" to clarify the way to recruit participants who were university students reachable in a closed university network. The word "web-based" and "self-assessed" were used to clarify that the study was a purely web-based trial and outcomes were self-assessed through questionnaires.

1b-iv) RESULTS section in abstract must contain use data

1b-v) CONCLUSIONS/DISCUSSION in abstract for negative trials

INTRODUCTION

2a-i) Problem and the type of system/solution

Although game-based learning in classroom setting was widely evaluated, the effectiveness of game-based learning on social networks is not yet fully evaluated. It is believed that social network game is possible to highly engage young people and equip them with mental health literacy. Although different type of web-based programs for mental health education were evaluated, the use of social network game for mental health education however was not explored and evaluated. The need of high-quality and interactive web-based programs for mental health education exists.

2a-ii) Scientific background, rationale: What is known about the (type of) system

The use of similar systems for other conditions: "“The Little Prince is Depressed” project was a 12-week, school-based universal programme aimed at reducing depressive symptoms and enhancing protective factors of depression among secondary school students. Its curriculum was developed based on cognitive-behavioural model and included topics like stress and depression, cognitive restructuring skills and problem-solving skills. Results indicated that students showed positive development in help-seeking attitudes and self-esteem in general and students with more depressive symptoms had significantly improved their cognitive restructuring skills and support-seeking behaviours. However, the number of beneficiaries of school-based programme would be limited to the resources available and competition of school teaching hours to carry out such programme is keen."

What are the reasons for: "the effectiveness of DGBL in mental health education has been underexplored. Taken together the findings and benefits of DGBL in health education, it is worthwhile to examine the effects of DGBL on mental health education"

What is the context for this specific study: "the effectiveness of DGBL in mental health education has been underexplored. Taken together the findings and benefits of DGBL in health education, it is worthwhile to examine the effects of DGBL on mental health education"

From which stakeholder viewpoint is the study performed: From educators and researchers' point of view. We saw a large demand of online health information and the opportunity of using advanced technology for mental health education. "some studies indicated that the Internet has become an instrumental information-searching tool for people with health concerns [1, 2], especially for adolescents [3, 4]. Informative educational websites on physical and mental health information Informative educational websites on physical and mental health information such as National Institutes of Health (http://health.nih.gov/), Beason (http://www.beacon.anu.edu.au/), beyondblue (http://www.beyondblue.org.au/) are some good examples of contemporary health education initiatives. The Internet has been proposed as an efficient platform to engage, educate, and intervene the younger generation with health concerns, especially for those who have not been easily engaged by traditional methods [5, 6]. There are some interventions primarily focused on providing mental health education such as Kindertelefoon, YooMagazine and ReachOut. Those interventions engage young people through interactive forums, games and websites to enhance their mental health literacy."

Potential impact of findings: "We hypothesized that mental health literacy would be enhanced through web-based DGBL and three motivational constructs, namely expectancy, value, and affect, would influence the learning outcome." The study may contribute to the web-based intervention of mental health education.

METHODS

3a) CONSORT: Description of trial design (such as parallel, factorial) including allocation ratio

The objectives were specified that "Specifically, we aimed at:
(1)exploring the effectiveness of using DGBL in enhancing mental health literacy through a SNS;
(2)exploring the learning motivation in DGBL;
(3)examining whether gender plays a role in the intervention efficacy and learning motivation; and
(4)exploring the impact of the motivational constructs on mental health literacy."

3b) CONSORT: Important changes to methods after trial commencement (such as eligibility criteria), with reasons

No important change to methods after trial commencement in the study.

3b-i) Bug fixes, Downtimes, Content Changes

4a) CONSORT: Eligibility criteria for participants
The eligibility criteria for participants was shown in the ms "Students who were aged 17-25 years old, had adequate Internet literacy, and a Facebook account reachable in the local network were eligible to participate in the study."
4a-i) Computer / Internet literacy
The eligibility criteria for participants was shown in the ms "Students who were aged 17-25 years old, had adequate Internet literacy, and a Facebook account reachable in the local network were eligible to participate in the study."
4a-ii) Open vs. closed, web-based vs. face-to-face assessments:
The participants were recruited from a closed user group "An invitation email was sent to all undergraduate and postgraduate students (n = 22,260) to their university e-mail accounts to invite them for participation in the current study."
The participants were required to provide their university number and Facebook ID for verification to prevent multiple identities of a participant "students were asked to reply to the invitation email and provide their Facebook ID for verification".
4a-iii) Information giving during recruitment

4b) CONSORT: Settings and locations where the data were collected
The data were collected on the web through questionnaires and stored in a database locked in the university. "Questionnaire and game progress data were stored in MySQL database. The system was hosted on a computer server on the authors' host institution network."
4b-i) Report if outcomes were (self-)assessed through online questionnaires
The participants were asked to "complete a set of web-based, self-assessed questionnaire before and after finishing the game".
4b-ii) Report how institutional affiliations are displayed

5) CONSORT: Describe the interventions for each group with sufficient details to allow replication, including how and when they were actually administered
5-i) Mention names, credential, affiliations of the developers, sponsors, and owners

5-ii) Describe the history/development process

5-iii) Revisions and updating

5-iv) Quality assurance methods

5-v) Ensure replicability by publishing the source code, and/or providing screenshots/screen-capture video, and/or providing flowcharts of the algorithms used

5-vi) Digital preservation

5-vii) Access
In order to access the application, a Facebook account was required so "a Facebook account reachable in the local network" was one of the eligibility criteria. After that, "the game was accessible by the public at http://apps.facebook.com/mentalhealthgame/."
5-viii) Mode of delivery, features/functionalities/components of the intervention and comparator, and the theoretical framework
"The electronic game "Ching Ching Story" and its content were developed by the authors of the paper." The features of the intervention were described in the ms "Its content was developed based on a cognitive-behavioral therapeutic approach" and "the game adopted a problem-based narrative adventure approach". The game design and instructional framework was also clearly stated in the ms.
5-ix) Describe use parameters

5-x) Clarify the level of human involvement
"Technical support was provided by the research team in case participants encountered any technical problems while playing the game. Apart from this, no other intervention or support was provided."
5-xi) Report any prompts/reminders used
"no prompts or reminders were used in the game to trigger frequency of gameplay, etc."
5-xii) Describe any co-interventions (incl. training/support)
"Technical support was provided by the research team in case participants encountered any technical problems while playing the game. Apart from this, no other intervention or support was provided."
6a) CONSORT: Completely defined pre-specified primary and secondary outcome measures, including how and when they were assessed
"Two sets of web-based, self-assessed questionnaires were developed for measuring participants' mental health literacy (the primary outcome) and their learning motivation (the secondary outcome)." "The first set (pre-test) consisted of demographic information and self-developed questions on mental health literacy while the second set (post-test) consisted of the same questions for mental health literacy plus modified questions for learning motivation from Motivated Strategies for Learning Questionnaire (MSLQ)" The questions in the outcome measures were put in the Appendix in the ms.
6a-i) Online questionnaires: describe if they were validated for online use and apply CHERRIES items to describe how the questionnaires were designed/deployed

Design: "The pre-test–post-test design was used in the study to evaluate the fully automated web-based intervention", "Participants were recruited at a major university in Asia" and "Students who were aged 17-25 years old, had adequate Internet literacy, and a Facebook account reachable in the local network were eligible to participate in the study"

IRB approval: "Ethics approval was obtained before data collection from Human Research Ethics Committee for Non-Clinical Faculties of the authors' institution. Informed consent from each participant was obtained online before they started the game. Information and procedures of the study were provided in the informed consent form."

Recruitment process and description of the sample having access to the questionnaire: "Participants recruited from a closed online user group self-assessed" and "Technical support was provided by the research team in case participants encountered any technical problems while playing the game."

Survey administration: "The pre and post questionnaires were automatically pop up once the game started and ended correspondingly", "Those who completed the game and the online questionnaires would get cash compensation and enter a lucky draw for a tablet computer, supermarket coupons, and theme park tickets", "The list of items used is shown in the Appendix"

Response rates: "A total of 221 undergraduates agreed to participate in the study. 136 of them started the game and out of these 136, 127 completed the pre questionnaire. Other 9 did not fully complete the pre questionnaire due to technical problems such as early termination of questionnaire. Out of these 127, 73 completed both the pre and post questionnaires. All 127 participants were included in ITT analysis while only the data from those 73 participants were used for completer analysis and regression analysis."

Preventing multiple entries from the same individual: "Facebook ID was used as a user identifier for verification and avoidance of duplicate entries"

Analysis: "Drop-out participants who only completed questionnaires at pretest were handled by applying the technique of MI with 5 imputations for missing data"

6a-ii) Describe whether and how "use" (including intensity of use/dosage) was defined/measured/monitored

6a-iii) Describe whether, how, and when qualitative feedback from participants was obtained

6b) CONSORT: Any changes to trial outcomes after the trial commenced, with reasons

No change to trial outcomes after the trial commenced.

7a) CONSORT: How sample size was determined

7a-i) Describe whether and how expected attrition was taken into account when calculating the sample size

7b) CONSORT: When applicable, explanation of any interim analyses and stopping guidelines

No interim analysis and stopping guideline

8a) CONSORT: Method used to generate the random allocation sequence

All participants recruited were allocated to the intervention group.

8b) CONSORT: Type of randomisation; details of any restriction (such as blocking and block size)

All participants recruited were allocated to the intervention group.

9) CONSORT: Mechanism used to implement the random allocation sequence (such as sequentially numbered containers), describing any steps taken to conceal the sequence until interventions were assigned

All participants recruited were allocated to the intervention group.

10) CONSORT: Who generated the random allocation sequence, who enrolled participants, and who assigned participants to interventions

All participants recruited were allocated to the intervention group. No random allocation sequence was generated.

11a) CONSORT: Blinding - If done, who was blinded after assignment to interventions (for example, participants, care providers, those assessing outcomes) and how

11a-i) Specify who was blinded, and who wasn't

All participants recruited were allocated to the intervention group. No blinding was done.

11a-ii) Discuss e.g., whether participants knew which intervention was the "intervention of interest" and which one was the "comparator"

11b) CONSORT: If relevant, description of the similarity of interventions

Just one intervention in the study.

12a) CONSORT: Statistical methods used to compare groups for primary and secondary outcomes

"A within-subject Student's t-test was used to analyze the significance of knowledge enhancement while a Student's t-test for independent samples was harnessed to analyze gender differences." "Linear regression was employed for examining the relation between knowledge enhancement and motivational constructs."

12a-i) Imputation techniques to deal with attrition / missing values

"Any participants with missing data or dropped out from the study were not included in completer analysis. In addition to completer analysis, data was analyzed by intent-to-treat (ITT) analysis, using multiple imputation (MI) to deal with loss to follow-up. Drop-out participants who only completed questionnaires at pretest were handled by applying the technique of MI with 5 imputations for missing data. Missing values analysis was conducted to investigate whether data were missing completely at random (MCAR)."

12b) CONSORT: Methods for additional analyses, such as subgroup analyses and adjusted analyses

No additional analysis.

RESULTS

13a) CONSORT: For each group, the numbers of participants who were randomly assigned, received intended treatment, and were analysed for the primary outcome

All 73 participants recruited were assigned to intervention group. "73 participants were used for completer analysis and regression analysis."

13b) CONSORT: For each group, losses and exclusions after randomisation, together with reasons

"A total of 221 undergraduates agreed to participate in the study. 136 of them started the game and out of these 136, 127 completed the pre questionnaire. Other 9 did not fully complete the pre questionnaire due to technical problems such as early termination of questionnaire. Out of these 127, 73 completed both the pre and post questionnaires."

13b-i) Attrition diagram

14a) CONSORT: Dates defining the periods of recruitment and follow-up

"Participants were recruited from November to December 2011 at the authors' institution." No follow-up in the study.
14a-i) Indicate if critical "secular events" fell into the study period

14b) CONSORT: Why the trial ended or was stopped (early)
The trial did not end or was not stopped early.
15) CONSORT: A table showing baseline demographic and clinical characteristics for each group
"The sample incorporated 73 undergraduates (31 males and 42 females)."
"Descriptive statistics were used to illustrate the general picture of the subjects for each measurement."
"Table 1 presents the number of correct responses of participants on their mental health literacy before and after playing the game, as well as their improvement."
"Table 2 presents the descriptive statistics of the motivational subscales."
15-i) Report demographics associated with digital divide issues
"The sample incorporated 73 undergraduates (31 males and 42 females)."
"Students who were aged 17-25 years old, had adequate Internet literacy, and a Facebook account reachable in the local network were eligible to participate in the study."
16a) CONSORT: For each group, number of participants (denominator) included in each analysis and whether the analysis was by original assigned groups
16-i) Report multiple "denominators" and provide definitions
"A total of 221 undergraduates agreed to participate in the study. 136 of them started the game and out of these 136, 127 completed the pre questionnaire. Other 9 did not fully complete the pre questionnaire due to technical problems such as early termination of questionnaire. Out of these 127, 73 completed both the pre and post questionnaires."
"All 127 participants were included in ITT analysis while only the data from those 73 participants were used for completer analysis and regression analysis." Out of 127, 73 completed both the pre and post questionnaires. 54 only completed the pre questionnaire.
"ITT analysis shows the improvement between pre and posttest was statistically significant with moderate effect size (ES = 0.66). The result is consistent with the completer analysis (ES = 0.65)."
16-ii) Primary analysis should be intent-to-treat
"data was analyzed by intent-to-treat (ITT) analysis, using multiple imputation (MI)"
"Missing values analysis was conducted to investigate whether data were missing completely at random (MCAR)."
"non-completers were also included in ITT to provide more understanding on the intervention. Missing values analysis was conducted on the data and demonstrated that the hypothesis that the data are MCAR cannot be rejected, X2 Little = 3.75 (P = .15). ITT analysis shows the improvement between pre and posttest was statistically significant with moderate effect size (ES = 0.66). The result is consistent with the completer analysis (ES = 0.65)."
17a) CONSORT: For each primary and secondary outcome, results for each group, and the estimated effect size and its precision (such as 95% confidence interval)
"A within-subject Student's t-test was used to analyze the significance of knowledge enhancement while a Student's t-test for independent samples was harnessed to analyze gender differences."
"By fitting knowledge enhancement with motivational constructs, the coefficient and 95% confidence interval are presented."
17a-i) Presentation of process outcomes such as metrics of use and intensity of use

17b) CONSORT: For binary outcomes, presentation of both absolute and relative effect sizes is recommended
The mean and standard deviation of the knowledge enhancement were presented.
"By fitting knowledge enhancement with motivational constructs, the coefficient and 95% confidence interval are presented."
18) CONSORT: Results of any other analyses performed, including subgroup analyses and adjusted analyses, distinguishing pre-specified from exploratory
no other analysis performed.
18-i) Subgroup analysis of comparing only users

19) CONSORT: All important harms or unintended effects in each group
Generally no harms or unintended effects existed in the intervention group. One possible unintended effect was Internet addiction.
19-i) Include privacy breaches, technical problems

19-ii) Include qualitative feedback from participants or observations from staff/researchers

DISCUSSION

20) CONSORT: Trial limitations, addressing sources of potential bias, imprecision, multiplicity of analyses
20-i) Typical limitations in ehealth trials
"the social functions in the game may not be fully utilized as the number of players was not very large. Time for the number of players in the game to grow was also not adequate. Since interactivity of a social game is substantially influenced by its number of players, the game in this study therefore may not be as interactive as other social games for pure entertainment in the market."
21) CONSORT: Generalisability (external validity, applicability) of the trial findings
21-i) Generalizability to other populations

21-ii) Discuss if there were elements in the RCT that would be different in a routine application setting

22) CONSORT: Interpretation consistent with results, balancing benefits and harms, and considering other relevant evidence
22-i) Restate study questions and summarize the answers suggested by the data, starting with primary outcomes and process outcomes (use)
"For mental health literacy (primary outcome), the completer analysis demonstrated moderate effect size (ES = 0.65). The ITT analysis showed a consistent result with the completer analysis. No gender difference was found (P = .97). For learning motivation (secondary outcome), intrinsic goal orientation was highest (4.97 points out of 7) while test anxiety was lowest (3.34 points out of 7) among the six motivational subscales. No gender difference was found in all subscales (P > .10). Besides, self-efficacy for learning and performance positively influenced the learning outcome while text anxiety negatively affected the learning outcome. The findings were instrumental to:
(1)showing the effectiveness of using DGBL in enhancing mental health literacy through a SNS;
(2)demonstrating the learning motivation in DGBL;
(3)examining whether gender plays a role in the intervention efficacy and learning motivation; and
(4)showing the impact of the motivational constructs on mental health literacy."

22-ii) Highlight unanswered new questions, suggest future research

"Further studies are required with a larger sample size with a suitable control and on different age groups in order to have a more comprehensive understanding of the effects of this web-based electronic game on mental health education. In addition to augmenting the sample size, other possible future research direction includes investigating the effectiveness of social game education based on this study. Social games on SNS facilitate social interaction and communication, making them different from traditional online games. SNS could provide tools to embed the communication platforms (e. g., blogs, forums) into the games which may enhance the sharing and knowledge exchange among players substantially. These social elements can motivate players to learn and potentially foster the learning process. It is believed that DGBL should not be restricted to learning inside the game but also outside the game, for example, through discussing learning material on blogs or forums. The effectiveness of social game education needs further evaluation. Besides, the scope of study can be extended to different health issues such as mood and eating disorders. The DGBL therefore can be fully utilized and evaluated in different aspects in health education."

Other information

23) CONSORT: Registration number and name of trial registry

The trial is not registered at any trial registry.

24) CONSORT: Where the full trial protocol can be accessed, if available

no full trial protocol can be accessed.

25) CONSORT: Sources of funding and other support (such as supply of drugs), role of funders

"This research has been supported in part by the Health Care and Promotion Fund."

X26-i) Comment on ethics committee approval

x26-ii) Outline informed consent procedures

X26-iii) Safety and security procedures

X27-i) State the relation of the study team towards the system being evaluated

The authors declare no conflict of interest.
